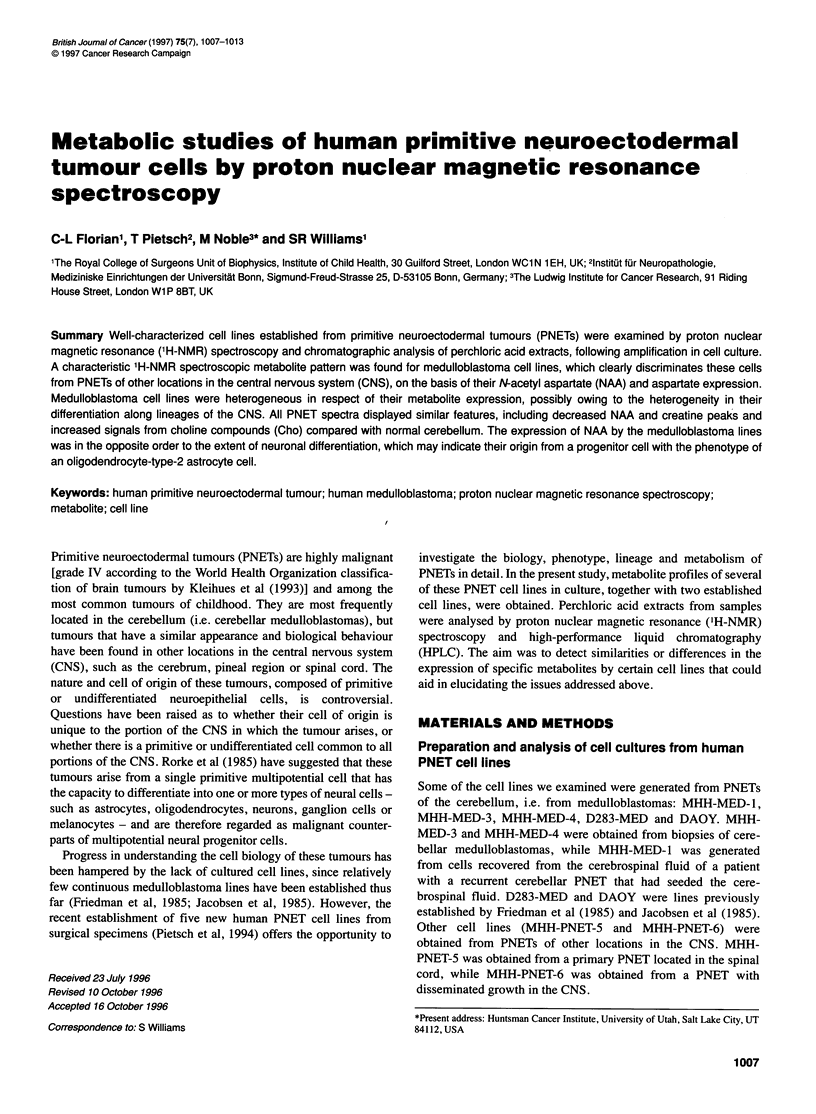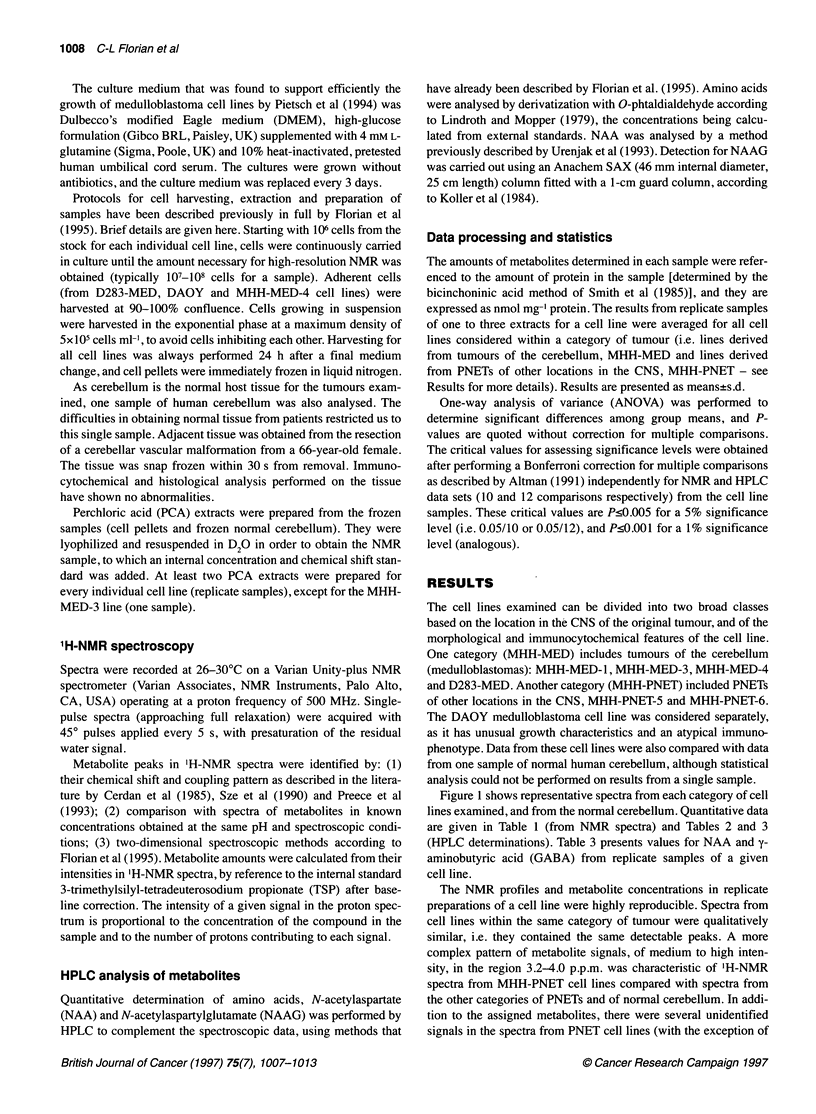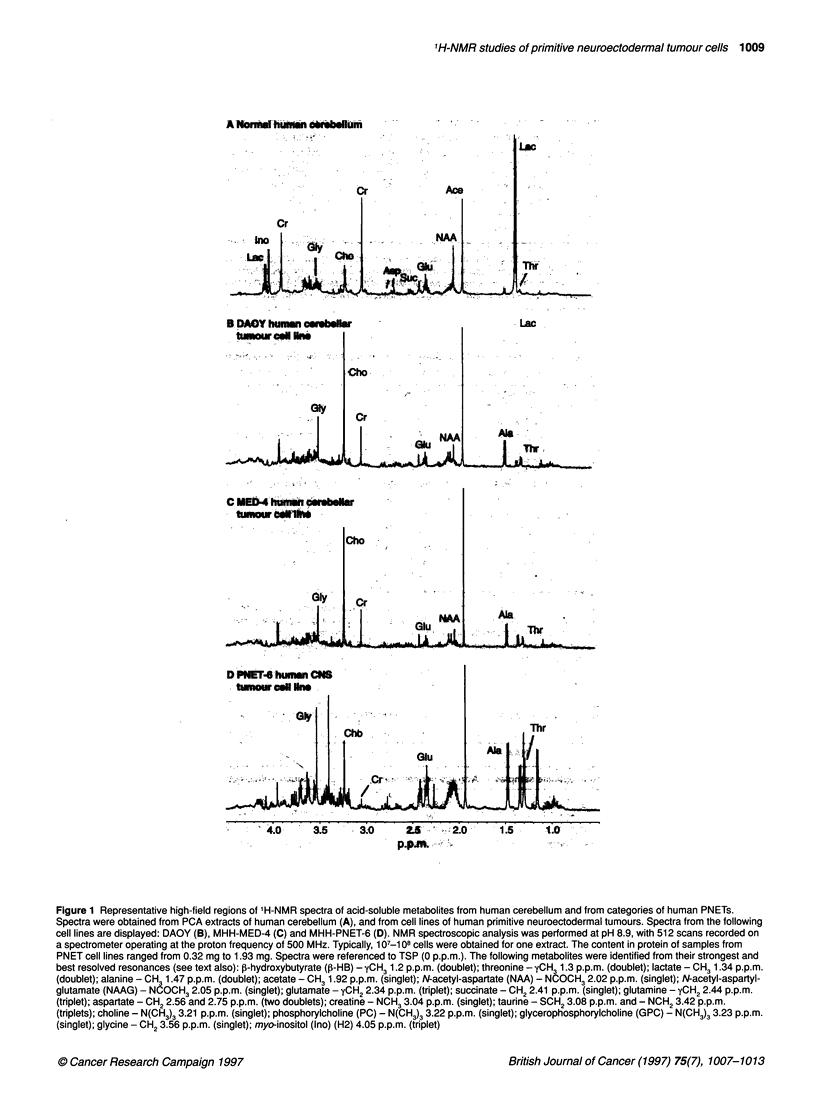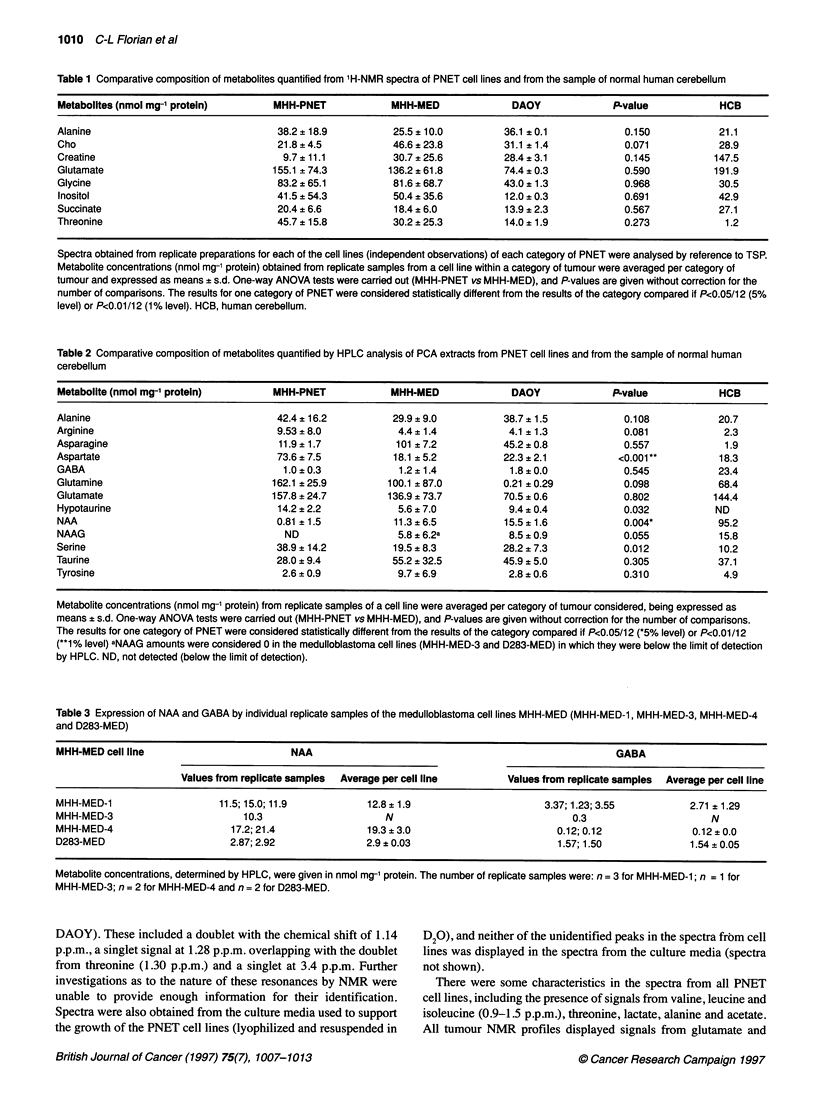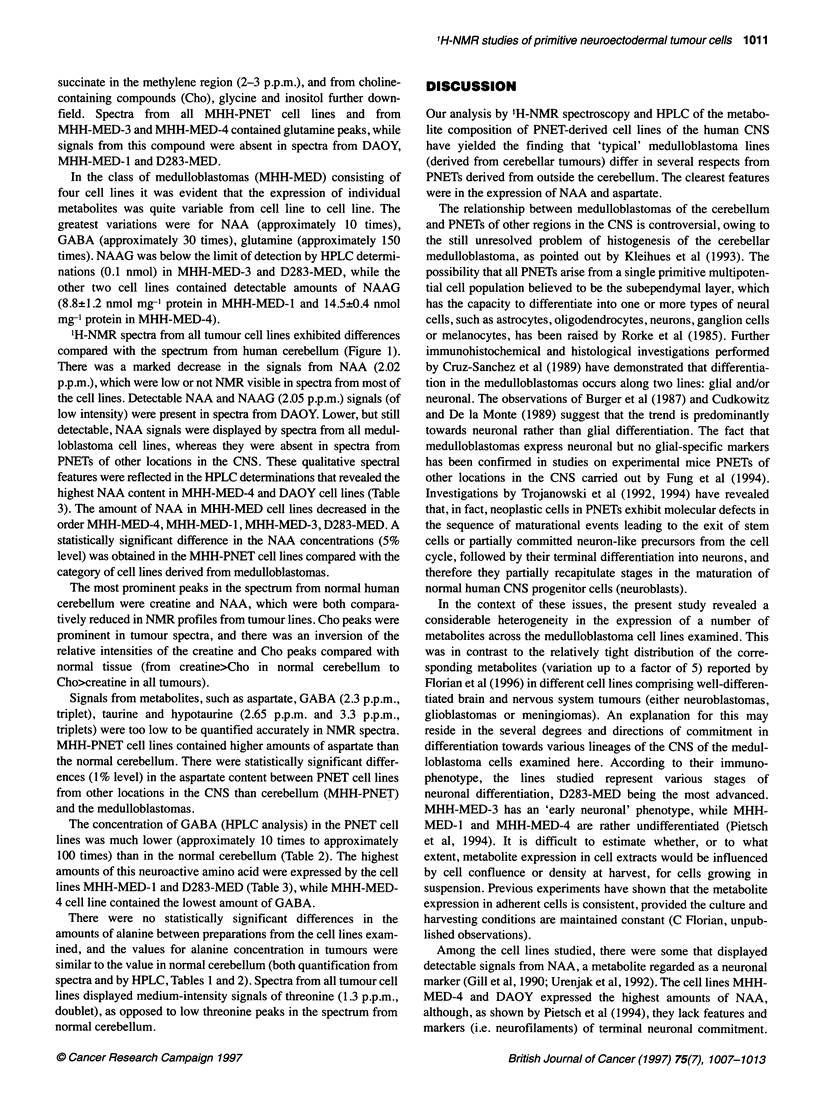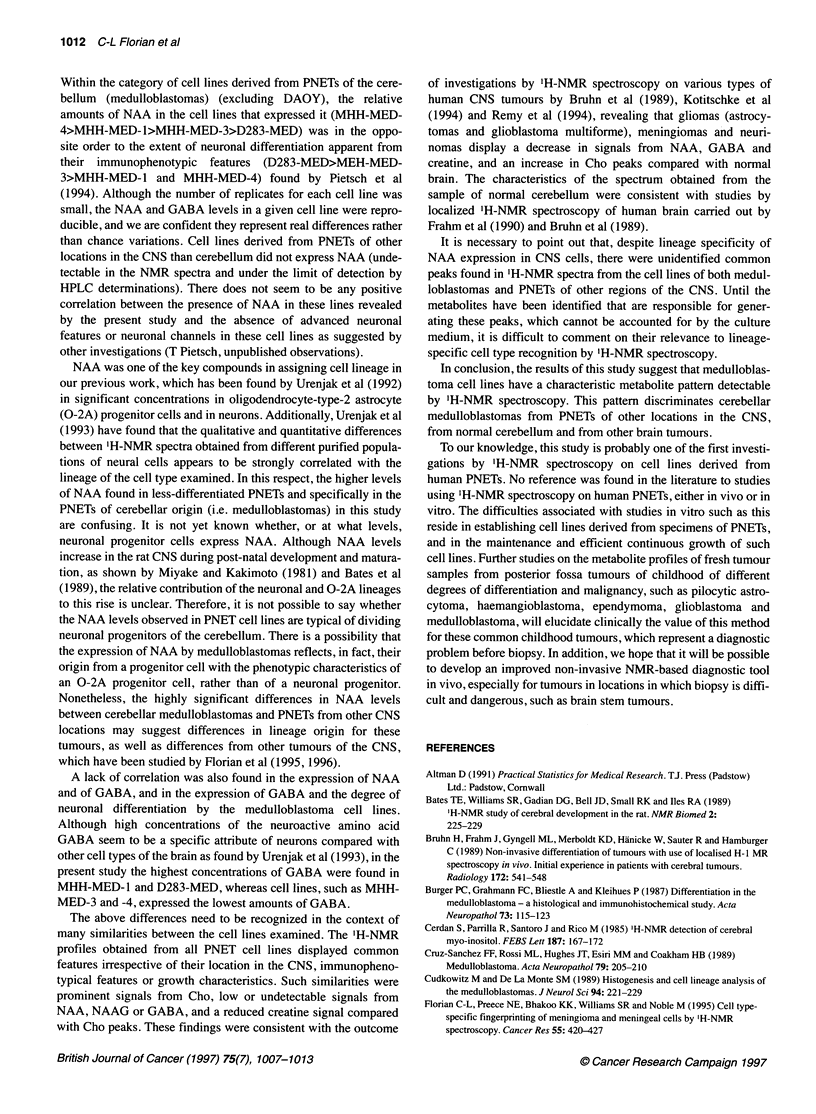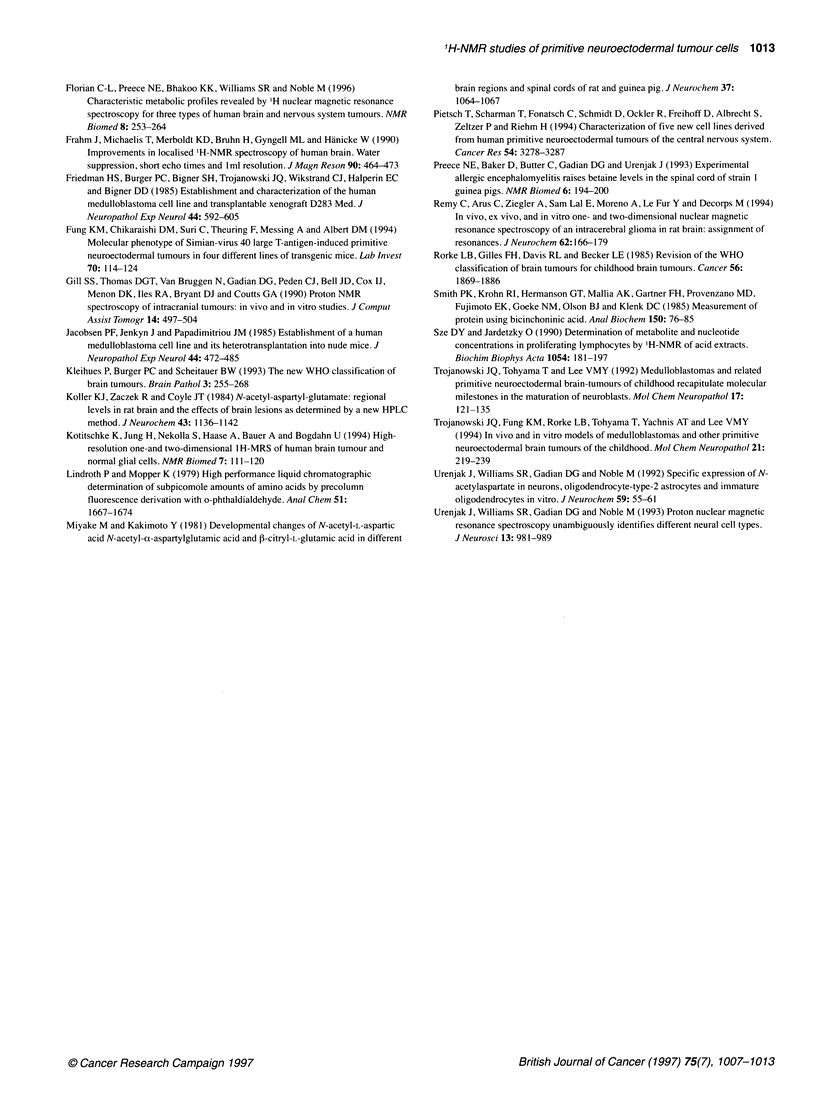# Metabolic studies of human primitive neuroectodermal tumour cells by proton nuclear magnetic resonance spectroscopy.

**DOI:** 10.1038/bjc.1997.173

**Published:** 1997

**Authors:** C. L. Florian, T. Pietsch, M. Noble, S. R. Williams

**Affiliations:** The Royal College of Surgeons Unit of Biophysics, Institute of Child Health, London, UK.

## Abstract

Well-characterized cell lines established from primitive neuroectodermal tumours (PNETs) were examined by proton nuclear magnetic resonance (1H-NMR) spectroscopy and chromatographic analysis of perchloric acid extracts, following amplification in cell culture. A characteristic 1H-NMR spectroscopic metabolite pattern was found for medulloblastoma cell lines, which clearly discriminates these cells from PNETs of other locations in the central nervous system (CNS), on the basis of their N-acetyl aspartate (NAA) and aspartate expression. Medulloblastoma cell lines were heterogeneous in respect of their metabolite expression, possibly owing to the heterogeneity in their differentiation along lineages of the CNS. All PNET spectra displayed similar features, including decreased NAA and creatine peaks and increased signals from choline compounds (Cho) compared with normal cerebellum. The expression of NAA by the medulloblastoma lines was in the opposite order to the extent of neuronal differentiation, which may indicate their origin from a progenitor cell with the phenotype of an oligodendrocyte-type-2 astrocyte cell.


*British Journal of Cancer* (1997) **75**(7), 1007–1013
© 1997 Cancer Research Campaign

### Metabolic studies of human primitive neuroectodermal tumour cells by proton nuclear magnetic resonance spectroscopy

**C-L Florian[1], T Pietsch[2], M Noble[3]* and SR Williams[1]**

[1]The Royal College of Surgeons Unit of Biophysics, Institute of Child Health, 30 Guilford Street, London WC1N 1EH, UK; [2]Institüt für Neuropathologie, Mediziniske Einrichtungen der Universität Bonn, Sigmund-Freud-Strasse 25, D-53105 Bonn, Germany; [3]The Ludwig Institute for Cancer Research, 91 Riding House Street, London W1P 8BT, UK

**Summary** Well-characterized cell lines established from primitive neuroectodermal tumours (PNETs) were examined by proton nuclear magnetic resonance ($^1$H-NMR) spectroscopy and chromatographic analysis of perchloric acid extracts, following amplification in cell culture. A characteristic $^1$H-NMR spectroscopic metabolite pattern was found for medulloblastoma cell lines, which clearly discriminates these cells from PNETs of other locations in the central nervous system (CNS), on the basis of their *N*-acetyl aspartate (NAA) and aspartate expression. Medulloblastoma cell lines were heterogeneous in respect of their metabolite expression, possibly owing to the heterogeneity in their differentiation along lineages of the CNS. All PNET spectra displayed similar features, including decreased NAA and creatine peaks and increased signals from choline compounds (Cho) compared with normal cerebellum. The expression of NAA by the medulloblastoma lines was in the opposite order to the extent of neuronal differentiation, which may indicate their origin from a progenitor cell with the phenotype of an oligodendrocyte-type-2 astrocyte cell.

**Keywords:** human primitive neuroectodermal tumour; human medulloblastoma; proton nuclear magnetic resonance spectroscopy; metabolite; cell line

Primitive neuroectodermal tumours (PNETs) are highly malignant [grade IV according to the World Health Organization classification of brain tumours by Kleihues et al (1993)] and among the most common tumours of childhood. They are most frequently located in the cerebellum (i.e. cerebellar medulloblastomas), but tumours that have a similar appearance and biological behaviour have been found in other locations in the central nervous system (CNS), such as the cerebrum, pineal region or spinal cord. The nature and cell of origin of these tumours, composed of primitive or undifferentiated neuroepithelial cells, is controversial. Questions have been raised as to whether their cell of origin is unique to the portion of the CNS in which the tumour arises, or whether there is a primitive or undifferentiated cell common to all portions of the CNS. Rorke et al (1985) have suggested that these tumours arise from a single primitive multipotential cell that has the capacity to differentiate into one or more types of neural cells – such as astrocytes, oligodendrocytes, neurons, ganglion cells or melanocytes – and are therefore regarded as malignant counterparts of multipotential neural progenitor cells.

Progress in understanding the cell biology of these tumours has been hampered by the lack of cultured cell lines, since relatively few continuous medulloblastoma lines have been established thus far (Friedman et al, 1985; Jacobsen et al, 1985). However, the recent establishment of five new human PNET cell lines from surgical specimens (Pietsch et al, 1994) offers the opportunity to investigate the biology, phenotype, lineage and metabolism of PNETs in detail. In the present study, metabolite profiles of several of these PNET cell lines in culture, together with two established cell lines, were obtained. Perchloric acid extracts from samples were analysed by proton nuclear magnetic resonance ($^1$H-NMR) spectroscopy and high-performance liquid chromatography (HPLC). The aim was to detect similarities or differences in the expression of specific metabolites by certain cell lines that could aid in elucidating the issues addressed above.

### MATERIALS AND METHODS

### Preparation and analysis of cell cultures from human PNET cell lines

Some of the cell lines we examined were generated from PNETs of the cerebellum, i.e. from medulloblastomas: MHH-MED-1, MHH-MED-3, MHH-MED-4, D283-MED and DAOY. MHH-MED-3 and MHH-MED-4 were obtained from biopsies of cerebellar medulloblastomas, while MHH-MED-1 was generated from cells recovered from the cerebrospinal fluid of a patient with a recurrent cerebellar PNET that had seeded the cerebrospinal fluid. D283-MED and DAOY were lines previously established by Friedman et al (1985) and Jacobsen et al (1985). Other cell lines (MHH-PNET-5 and MHH-PNET-6) were obtained from PNETs of other locations in the CNS. MHH-PNET-5 was obtained from a primary PNET located in the spinal cord, while MHH-PNET-6 was obtained from a PNET with disseminated growth in the CNS.

*Received 23 July 1996*
*Revised 10 October 1996*
*Accepted 16 October 1996*

*Correspondence to:* S Williams

*Present address: Huntsman Cancer Institute, University of Utah, Salt Lake City, UT 84112, USA

The culture medium that was found to support efficiently the growth of medulloblastoma cell lines by Pietsch et al (1994) was Dulbecco's modified Eagle medium (DMEM), high-glucose formulation (Gibco BRL, Paisley, UK) supplemented with 4 mM L-glutamine (Sigma, Poole, UK) and 10% heat-inactivated, pretested human umbilical cord serum. The cultures were grown without antibiotics, and the culture medium was replaced every 3 days.

Protocols for cell harvesting, extraction and preparation of samples have been described previously in full by Florian et al (1995). Brief details are given here. Starting with $10^6$ cells from the stock for each individual cell line, cells were continuously carried in culture until the amount necessary for high-resolution NMR was obtained (typically $10^7$–$10^8$ cells for a sample). Adherent cells (from D283-MED, DAOY and MHH-MED-4 cell lines) were harvested at 90–100% confluence. Cells growing in suspension were harvested in the exponential phase at a maximum density of $5\times10^5$ cells $ml^{-1}$, to avoid cells inhibiting each other. Harvesting for all cell lines was always performed 24 h after a final medium change, and cell pellets were immediately frozen in liquid nitrogen.

As cerebellum is the normal host tissue for the tumours examined, one sample of human cerebellum was also analysed. The difficulties in obtaining normal tissue from patients restricted us to this single sample. Adjacent tissue was obtained from the resection of a cerebellar vascular malformation from a 66-year-old female. The tissue was snap frozen within 30 s from removal. Immunocytochemical and histological analysis performed on the tissue have shown no abnormalities.

Perchloric acid (PCA) extracts were prepared from the frozen samples (cell pellets and frozen normal cerebellum). They were lyophilized and resuspended in $D_2O$ in order to obtain the NMR sample, to which an internal concentration and chemical shift standard was added. At least two PCA extracts were prepared for every individual cell line (replicate samples), except for the MHH-MED-3 line (one sample).

### $^1$H-NMR spectroscopy

Spectra were recorded at 26–30°C on a Varian Unity-plus NMR spectrometer (Varian Associates, NMR Instruments, Palo Alto, CA, USA) operating at a proton frequency of 500 MHz. Single-pulse spectra (approaching full relaxation) were acquired with 45° pulses applied every 5 s, with presaturation of the residual water signal.

Metabolite peaks in $^1$H-NMR spectra were identified by: (1) their chemical shift and coupling pattern as described in the literature by Cerdan et al (1985), Sze et al (1990) and Preece et al (1993); (2) comparison with spectra of metabolites in known concentrations obtained at the same pH and spectroscopic conditions; (3) two-dimensional spectroscopic methods according to Florian et al (1995). Metabolite amounts were calculated from their intensities in $^1$H-NMR spectra, by reference to the internal standard 3-trimethylsilyl-tetradeuterosodium propionate (TSP) after baseline correction. The intensity of a given signal in the proton spectrum is proportional to the concentration of the compound in the sample and to the number of protons contributing to each signal.

### HPLC analysis of metabolites

Quantitative determination of amino acids, *N*-acetylaspartate (NAA) and *N*-acetylaspartylglutamate (NAAG) was performed by HPLC to complement the spectroscopic data, using methods that have already been described by Florian et al. (1995). Amino acids were analysed by derivatization with *O*-phtaldialdehyde according to Lindroth and Mopper (1979), the concentrations being calculated from external standards. NAA was analysed by a method previously described by Urenjak et al (1993). Detection for NAAG was carried out using an Anachem SAX (46 mm internal diameter, 25 cm length) column fitted with a 1-cm guard column, according to Koller et al (1984).

### Data processing and statistics

The amounts of metabolites determined in each sample were referenced to the amount of protein in the sample [determined by the bicinchoninic acid method of Smith et al (1985)], and they are expressed as nmol $mg^{-1}$ protein. The results from replicate samples of one to three extracts for a cell line were averaged for all cell lines considered within a category of tumour (i.e. lines derived from tumours of the cerebellum, MHH-MED and lines derived from PNETs of other locations in the CNS, MHH-PNET – see Results for more details). Results are presented as means±s.d.

One-way analysis of variance (ANOVA) was performed to determine significant differences among group means, and *P*-values are quoted without correction for multiple comparisons. The critical values for assessing significance levels were obtained after performing a Bonferroni correction for multiple comparisons as described by Altman (1991) independently for NMR and HPLC data sets (10 and 12 comparisons respectively) from the cell line samples. These critical values are $P\leq0.005$ for a 5% significance level (i.e. 0.05/10 or 0.05/12), and $P\leq0.001$ for a 1% significance level (analogous).

### RESULTS

The cell lines examined can be divided into two broad classes based on the location in the CNS of the original tumour, and of the morphological and immunocytochemical features of the cell line. One category (MHH-MED) includes tumours of the cerebellum (medulloblastomas): MHH-MED-1, MHH-MED-3, MHH-MED-4 and D283-MED. Another category (MHH-PNET) included PNETs of other locations in the CNS, MHH-PNET-5 and MHH-PNET-6. The DAOY medulloblastoma cell line was considered separately, as it has unusual growth characteristics and an atypical immunophenotype. Data from these cell lines were also compared with data from one sample of normal human cerebellum, although statistical analysis could not be performed on results from a single sample.

Figure 1 shows representative spectra from each category of cell lines examined, and from the normal cerebellum. Quantitative data are given in Table 1 (from NMR spectra) and Tables 2 and 3 (HPLC determinations). Table 3 presents values for NAA and γ-aminobutyric acid (GABA) from replicate samples of a given cell line.

The NMR profiles and metabolite concentrations in replicate preparations of a cell line were highly reproducible. Spectra from cell lines within the same category of tumour were qualitatively similar, i.e. they contained the same detectable peaks. A more complex pattern of metabolite signals, of medium to high intensity, in the region 3.2–4.0 p.p.m. was characteristic of $^1$H-NMR spectra from MHH-PNET cell lines compared with spectra from the other categories of PNETs and of normal cerebellum. In addition to the assigned metabolites, there were several unidentified signals in the spectra from PNET cell lines (with the exception of

 © Cancer Research Campaign 1997

**Figure 1** Representative high-field regions of $^{1}$H-NMR spectra of acid-soluble metabolites from human cerebellum and from categories of human PNETs. Spectra were obtained from PCA extracts of human cerebellum (**A**), and from cell lines of human primitive neuroectodermal tumours. Spectra from the following cell lines are displayed: DAOY (**B**), MHH-MED-4 (**C**) and MHH-PNET-6 (**D**). NMR spectroscopic analysis was performed at pH 8.9, with 512 scans recorded on a spectrometer operating at the proton frequency of 500 MHz. Typically, $10^7$–$10^8$ cells were obtained for one extract. The content in protein of samples from PNET cell lines ranged from 0.32 mg to 1.93 mg. Spectra were referenced to TSP (0 p.p.m.). The following metabolites were identified from their strongest and best resolved resonances (see text also): β-hydroxybutyrate (β-HB) – $\gamma CH_3$ 1.2 p.p.m. (doublet); threonine – $\gamma CH_3$ 1.3 p.p.m. (doublet); lactate – $CH_3$ 1.34 p.p.m. (doublet); alanine – $CH_3$ 1.47 p.p.m. (doublet); acetate – $CH_3$ 1.92 p.p.m. (singlet); *N*-acetyl-aspartate (NAA) – $NCOCH_3$ 2.02 p.p.m. (singlet); *N*-acetyl-aspartyl-glutamate (NAAG) – $NCOCH_3$ 2.05 p.p.m. (singlet); glutamate – $\gamma CH_2$ 2.34 p.p.m. (triplet); succinate – $CH_2$ 2.41 p.p.m. (singlet); glutamine – $\gamma CH_2$ 2.44 p.p.m. (triplet); aspartate – $CH_2$ 2.56 and 2.75 p.p.m. (two doublets); creatine – $NCH_3$ 3.04 p.p.m. (singlet); taurine – $SCH_2$ 3.08 p.p.m. and – $NCH_2$ 3.42 p.p.m. (triplets); choline – $N(CH_3)_3$ 3.21 p.p.m. (singlet); phosphorylcholine (PC) – $N(CH_3)_3$ 3.22 p.p.m. (singlet); glycerophosphorylcholine (GPC) – $N(CH_3)_3$ 3.23 p.p.m. (singlet); glycine – $CH_2$ 3.56 p.p.m. (singlet); *myo*-inositol (Ino) (H2) 4.05 p.p.m. (triplet)

© Cancer Research Campaign 1997 

**Table 1** Comparative composition of metabolites quantified from ¹H-NMR spectra of PNET cell lines and from the sample of normal human cerebellum

| Metabolites (nmol $mg^{-1}$ protein) | MHH-PNET | MHH-MED | DAOY | *P*-value | HCB |
|---|---|---|---|---|---|
| Alanine | 38.2 ± 18.9 | 25.5 ± 10.0 | 36.1 ± 0.1 | 0.150 | 21.1 |
| Cho | 21.8 ± 4.5 | 46.6 ± 23.8 | 31.1 ± 1.4 | 0.071 | 28.9 |
| Creatine | 9.7 ± 11.1 | 30.7 ± 25.6 | 28.4 ± 3.1 | 0.145 | 147.5 |
| Glutamate | 155.1 ± 74.3 | 136.2 ± 61.8 | 74.4 ± 0.3 | 0.590 | 191.9 |
| Glycine | 83.2 ± 65.1 | 81.6 ± 68.7 | 43.0 ± 1.3 | 0.968 | 30.5 |
| Inositol | 41.5 ± 54.3 | 50.4 ± 35.6 | 12.0 ± 0.3 | 0.691 | 42.9 |
| Succinate | 20.4 ± 6.6 | 18.4 ± 6.0 | 13.9 ± 2.3 | 0.567 | 27.1 |
| Threonine | 45.7 ± 15.8 | 30.2 ± 25.3 | 14.0 ± 1.9 | 0.273 | 1.2 |

Spectra obtained from replicate preparations for each of the cell lines (independent observations) of each category of PNET were analysed by reference to TSP. Metabolite concentrations (nmol $mg^{-1}$ protein) obtained from replicate samples from a cell line within a category of tumour were averaged per category of tumour and expressed as means ± s.d. One-way ANOVA tests were carried out (MHH-PNET *vs* MHH-MED), and *P*-values are given without correction for the number of comparisons. The results for one category of PNET were considered statistically different from the results of the category compared if $P<0.05/12$ (5% level) or $P<0.01/12$ (1% level). HCB, human cerebellum.

**Table 2** Comparative composition of metabolites quantified by HPLC analysis of PCA extracts from PNET cell lines and from the sample of normal human cerebellum

| Metabolite (nmol $mg^{-1}$ protein) | MHH-PNET | MHH-MED | DAOY | *P*-value | HCB |
|---|---|---|---|---|---|
| Alanine | 42.4 ± 16.2 | 29.9 ± 9.0 | 38.7 ± 1.5 | 0.108 | 20.7 |
| Arginine | 9.53 ± 8.0 | 4.4 ± 1.4 | 4.1 ± 1.3 | 0.081 | 2.3 |
| Asparagine | 11.9 ± 1.7 | 101 ± 7.2 | 45.2 ± 0.8 | 0.557 | 1.9 |
| Aspartate | 73.6 ± 7.5 | 18.1 ± 5.2 | 22.3 ± 2.1 | <0.001** | 18.3 |
| GABA | 1.0 ± 0.3 | 1.2 ± 1.4 | 1.8 ± 0.0 | 0.545 | 23.4 |
| Glutamine | 162.1 ± 25.9 | 100.1 ± 87.0 | 0.21 ± 0.29 | 0.098 | 68.4 |
| Glutamate | 157.8 ± 24.7 | 136.9 ± 73.7 | 70.5 ± 0.6 | 0.802 | 144.4 |
| Hypotaurine | 14.2 ± 2.2 | 5.6 ± 7.0 | 9.4 ± 0.4 | 0.032 | ND |
| NAA | 0.81 ± 1.5 | 11.3 ± 6.5 | 15.5 ± 1.6 | 0.004* | 95.2 |
| NAAG | ND | 5.8 ± 6.2[a] | 8.5 ± 0.9 | 0.055 | 15.8 |
| Serine | 38.9 ± 14.2 | 19.5 ± 8.3 | 28.2 ± 7.3 | 0.012 | 10.2 |
| Taurine | 28.0 ± 9.4 | 55.2 ± 32.5 | 45.9 ± 5.0 | 0.305 | 37.1 |
| Tyrosine | 2.6 ± 0.9 | 9.7 ± 6.9 | 2.8 ± 0.6 | 0.310 | 4.9 |

Metabolite concentrations (nmol $mg^{-1}$ protein) from replicate samples of a cell line were averaged per category of tumour considered, being expressed as means ± s.d. One-way ANOVA tests were carried out (MHH-PNET *vs* MHH-MED), and *P*-values are given without correction for the number of comparisons. The results for one category of PNET were considered statistically different from the results of the category compared if $P<0.05/12$ (*5% level) or $P<0.01/12$ (**1% level) [a]NAAG amounts were considered 0 in the medulloblastoma cell lines (MHH-MED-3 and D283-MED) in which they were below the limit of detection by HPLC. ND, not detected (below the limit of detection).

**Table 3** Expression of NAA and GABA by individual replicate samples of the medulloblastoma cell lines MHH-MED (MHH-MED-1, MHH-MED-3, MHH-MED-4 and D283-MED)

| MHH-MED cell line | NAA | | GABA | |
|---|---|---|---|---|
| | Values from replicate samples | Average per cell line | Values from replicate samples | Average per cell line |
| MHH-MED-1 | 11.5; 15.0; 11.9 | 12.8 ± 1.9 | 3.37; 1.23; 3.55 | 2.71 ± 1.29 |
| MHH-MED-3 | 10.3 | *N* | 0.3 | *N* |
| MHH-MED-4 | 17.2; 21.4 | 19.3 ± 3.0 | 0.12; 0.12 | 0.12 ± 0.0 |
| D283-MED | 2.87; 2.92 | 2.9 ± 0.03 | 1.57; 1.50 | 1.54 ± 0.05 |

Metabolite concentrations, determined by HPLC, were given in nmol $mg^{-1}$ protein. The number of replicate samples were: $n = 3$ for MHH-MED-1; $n = 1$ for MHH-MED-3; $n = 2$ for MHH-MED-4 and $n = 2$ for D283-MED.

DAOY). These included a doublet with the chemical shift of 1.14 p.p.m., a singlet signal at 1.28 p.p.m. overlapping with the doublet from threonine (1.30 p.p.m.) and a singlet at 3.4 p.p.m. Further investigations as to the nature of these resonances by NMR were unable to provide enough information for their identification. Spectra were also obtained from the culture media used to support the growth of the PNET cell lines (lyophilized and resuspended in $D_2O$), and neither of the unidentified peaks in the spectra from cell lines was displayed in the spectra from the culture media (spectra not shown).

There were some characteristics in the spectra from all PNET cell lines, including the presence of signals from valine, leucine and isoleucine (0.9–1.5 p.p.m.), threonine, lactate, alanine and acetate. All tumour NMR profiles displayed signals from glutamate and

© Cancer Research Campaign 1997

succinate in the methylene region (2–3 p.p.m.), and from choline-containing compounds (Cho), glycine and inositol further downfield. Spectra from all MHH-PNET cell lines and from MHH-MED-3 and MHH-MED-4 contained glutamine peaks, while signals from this compound were absent in spectra from DAOY, MHH-MED-1 and D283-MED.

In the class of medulloblastomas (MHH-MED) consisting of four cell lines it was evident that the expression of individual metabolites was quite variable from cell line to cell line. The greatest variations were for NAA (approximately 10 times), GABA (approximately 30 times), glutamine (approximately 150 times). NAAG was below the limit of detection by HPLC determinations (0.1 nmol) in MHH-MED-3 and D283-MED, while the other two cell lines contained detectable amounts of NAAG (8.8±1.2 nmol $mg^{-1}$ protein in MHH-MED-1 and 14.5±0.4 nmol $mg^{-1}$ protein in MHH-MED-4).

$^1$H-NMR spectra from all tumour cell lines exhibited differences compared with the spectrum from human cerebellum (Figure 1). There was a marked decrease in the signals from NAA (2.02 p.p.m.), which were low or not NMR visible in spectra from most of the cell lines. Detectable NAA and NAAG (2.05 p.p.m.) signals (of low intensity) were present in spectra from DAOY. Lower, but still detectable, NAA signals were displayed by spectra from all medulloblastoma cell lines, whereas they were absent in spectra from PNETs of other locations in the CNS. These qualitative spectral features were reflected in the HPLC determinations that revealed the highest NAA content in MHH-MED-4 and DAOY cell lines (Table 3). The amount of NAA in MHH-MED cell lines decreased in the order MHH-MED-4, MHH-MED-1, MHH-MED-3, D283-MED. A statistically significant difference in the NAA concentrations (5% level) was obtained in the MHH-PNET cell lines compared with the category of cell lines derived from medulloblastomas.

The most prominent peaks in the spectrum from normal human cerebellum were creatine and NAA, which were both comparatively reduced in NMR profiles from tumour lines. Cho peaks were prominent in tumour spectra, and there was an inversion of the relative intensities of the creatine and Cho peaks compared with normal tissue (from creatine>Cho in normal cerebellum to Cho>creatine in all tumours).

Signals from metabolites, such as aspartate, GABA (2.3 p.p.m., triplet), taurine and hypotaurine (2.65 p.p.m. and 3.3 p.p.m., triplets) were too low to be quantified accurately in NMR spectra. MHH-PNET cell lines contained higher amounts of aspartate than the normal cerebellum. There were statistically significant differences (1% level) in the aspartate content between PNET cell lines from other locations in the CNS than cerebellum (MHH-PNET) and the medulloblastomas.

The concentration of GABA (HPLC analysis) in the PNET cell lines was much lower (approximately 10 times to approximately 100 times) than in the normal cerebellum (Table 2). The highest amounts of this neuroactive amino acid were expressed by the cell lines MHH-MED-1 and D283-MED (Table 3), while MHH-MED-4 cell line contained the lowest amount of GABA.

There were no statistically significant differences in the amounts of alanine between preparations from the cell lines examined, and the values for alanine concentration in tumours were similar to the value in normal cerebellum (both quantification from spectra and by HPLC, Tables 1 and 2). Spectra from all tumour cell lines displayed medium-intensity signals of threonine (1.3 p.p.m., doublet), as opposed to low threonine peaks in the spectrum from normal cerebellum.

### DISCUSSION

Our analysis by $^1$H-NMR spectroscopy and HPLC of the metabolite composition of PNET-derived cell lines of the human CNS have yielded the finding that 'typical' medulloblastoma lines (derived from cerebellar tumours) differ in several respects from PNETs derived from outside the cerebellum. The clearest features were in the expression of NAA and aspartate.

The relationship between medulloblastomas of the cerebellum and PNETs of other regions in the CNS is controversial, owing to the still unresolved problem of histogenesis of the cerebellar medulloblastoma, as pointed out by Kleihues et al (1993). The possibility that all PNETs arise from a single primitive multipotential cell population believed to be the subependymal layer, which has the capacity to differentiate into one or more types of neural cells, such as astrocytes, oligodendrocytes, neurons, ganglion cells or melanocytes, has been raised by Rorke et al (1985). Further immunohistochemical and histological investigations performed by Cruz-Sanchez et al (1989) have demonstrated that differentiation in the medulloblastomas occurs along two lines: glial and/or neuronal. The observations of Burger et al (1987) and Cudkowitz and De la Monte (1989) suggest that the trend is predominantly towards neuronal rather than glial differentiation. The fact that medulloblastomas express neuronal but no glial-specific markers has been confirmed in studies on experimental mice PNETs of other locations in the CNS carried out by Fung et al (1994). Investigations by Trojanowski et al (1992, 1994) have revealed that, in fact, neoplastic cells in PNETs exhibit molecular defects in the sequence of maturational events leading to the exit of stem cells or partially committed neuron-like precursors from the cell cycle, followed by their terminal differentiation into neurons, and therefore they partially recapitulate stages in the maturation of normal human CNS progenitor cells (neuroblasts).

In the context of these issues, the present study revealed a considerable heterogeneity in the expression of a number of metabolites across the medulloblastoma cell lines examined. This was in contrast to the relatively tight distribution of the corresponding metabolites (variation up to a factor of 5) reported by Florian et al (1996) in different cell lines comprising well-differentiated brain and nervous system tumours (either neuroblastomas, glioblastomas or meningiomas). An explanation for this may reside in the several degrees and directions of commitment in differentiation towards various lineages of the CNS of the medulloblastoma cells examined here. According to their immunophenotype, the lines studied represent various stages of neuronal differentiation, D283-MED being the most advanced. MHH-MED-3 has an 'early neuronal' phenotype, while MHH-MED-1 and MHH-MED-4 are rather undifferentiated (Pietsch et al, 1994). It is difficult to estimate whether, or to what extent, metabolite expression in cell extracts would be influenced by cell confluence or density at harvest, for cells growing in suspension. Previous experiments have shown that the metabolite expression in adherent cells is consistent, provided the culture and harvesting conditions are maintained constant (C Florian, unpublished observations).

Among the cell lines studied, there were some that displayed detectable signals from NAA, a metabolite regarded as a neuronal marker (Gill et al, 1990; Urenjak et al, 1992). The cell lines MHH-MED-4 and DAOY expressed the highest amounts of NAA, although, as shown by Pietsch et al (1994), they lack features and markers (i.e. neurofilaments) of terminal neuronal commitment.

© Cancer Research Campaign 1997

Within the category of cell lines derived from PNETs of the cerebellum (medulloblastomas) (excluding DAOY), the relative amounts of NAA in the cell lines that expressed it (MHH-MED-4>MHH-MED-1>MHH-MED-3>D283-MED) was in the opposite order to the extent of neuronal differentiation apparent from their immunophenotypic features (D283-MED>MEH-MED-3>MHH-MED-1 and MHH-MED-4) found by Pietsch et al (1994). Although the number of replicates for each cell line was small, the NAA and GABA levels in a given cell line were reproducible, and we are confident they represent real differences rather than chance variations. Cell lines derived from PNETs of other locations in the CNS than cerebellum did not express NAA (undetectable in the NMR spectra and under the limit of detection by HPLC determinations). There does not seem to be any positive correlation between the presence of NAA in these lines revealed by the present study and the absence of advanced neuronal features or neuronal channels in these cell lines as suggested by other investigations (T Pietsch, unpublished observations).

NAA was one of the key compounds in assigning cell lineage in our previous work, which has been found by Urenjak et al (1992) in significant concentrations in oligodendrocyte-type-2 astrocyte (O-2A) progenitor cells and in neurons. Additionally, Urenjak et al (1993) have found that the qualitative and quantitative differences between $^{1}$H-NMR spectra obtained from different purified populations of neural cells appears to be strongly correlated with the lineage of the cell type examined. In this respect, the higher levels of NAA found in less-differentiated PNETs and specifically in the PNETs of cerebellar origin (i.e. medulloblastomas) in this study are confusing. It is not yet known whether, or at what levels, neuronal progenitor cells express NAA. Although NAA levels increase in the rat CNS during post-natal development and maturation, as shown by Miyake and Kakimoto (1981) and Bates et al (1989), the relative contribution of the neuronal and O-2A lineages to this rise is unclear. Therefore, it is not possible to say whether the NAA levels observed in PNET cell lines are typical of dividing neuronal progenitors of the cerebellum. There is a possibility that the expression of NAA by medulloblastomas reflects, in fact, their origin from a progenitor cell with the phenotypic characteristics of an O-2A progenitor cell, rather than of a neuronal progenitor. Nonetheless, the highly significant differences in NAA levels between cerebellar medulloblastomas and PNETs from other CNS locations may suggest differences in lineage origin for these tumours, as well as differences from other tumours of the CNS, which have been studied by Florian et al (1995, 1996).

A lack of correlation was also found in the expression of NAA and of GABA, and in the expression of GABA and the degree of neuronal differentiation by the medulloblastoma cell lines. Although high concentrations of the neuroactive amino acid GABA seem to be a specific attribute of neurons compared with other cell types of the brain as found by Urenjak et al (1993), in the present study the highest concentrations of GABA were found in MHH-MED-1 and D283-MED, whereas cell lines, such as MHH-MED-3 and -4, expressed the lowest amounts of GABA.

The above differences need to be recognized in the context of many similarities between the cell lines examined. The $^{1}$H-NMR profiles obtained from all PNET cell lines displayed common features irrespective of their location in the CNS, immunophenotypical features or growth characteristics. Such similarities were prominent signals from Cho, low or undetectable signals from NAA, NAAG or GABA, and a reduced creatine signal compared with Cho peaks. These findings were consistent with the outcome of investigations by $^{1}$H-NMR spectroscopy on various types of human CNS tumours by Bruhn et al (1989), Kotitschke et al (1994) and Remy et al (1994), revealing that gliomas (astrocytomas and glioblastoma multiforme), meningiomas and neurinomas display a decrease in signals from NAA, GABA and creatine, and an increase in Cho peaks compared with normal brain. The characteristics of the spectrum obtained from the sample of normal cerebellum were consistent with studies by localized $^{1}$H-NMR spectroscopy of human brain carried out by Frahm et al (1990) and Bruhn et al (1989).

It is necessary to point out that, despite lineage specificity of NAA expression in CNS cells, there were unidentified common peaks found in $^{1}$H-NMR spectra from the cell lines of both medulloblastomas and PNETs of other regions of the CNS. Until the metabolites have been identified that are responsible for generating these peaks, which cannot be accounted for by the culture medium, it is difficult to comment on their relevance to lineage-specific cell type recognition by $^{1}$H-NMR spectroscopy.

In conclusion, the results of this study suggest that medulloblastoma cell lines have a characteristic metabolite pattern detectable by $^{1}$H-NMR spectroscopy. This pattern discriminates cerebellar medulloblastomas from PNETs of other locations in the CNS, from normal cerebellum and from other brain tumours.

To our knowledge, this study is probably one of the first investigations by $^{1}$H-NMR spectroscopy on cell lines derived from human PNETs. No reference was found in the literature to studies using $^{1}$H-NMR spectroscopy on human PNETs, either in vivo or in vitro. The difficulties associated with studies in vitro such as this reside in establishing cell lines derived from specimens of PNETs, and in the maintenance and efficient continuous growth of such cell lines. Further studies on the metabolite profiles of fresh tumour samples from posterior fossa tumours of childhood of different degrees of differentiation and malignancy, such as pilocytic astrocytoma, haemangioblastoma, ependymoma, glioblastoma and medulloblastoma, will elucidate clinically the value of this method for these common childhood tumours, which represent a diagnostic problem before biopsy. In addition, we hope that it will be possible to develop an improved non-invasive NMR-based diagnostic tool in vivo, especially for tumours in locations in which biopsy is difficult and dangerous, such as brain stem tumours.

### REFERENCES

Altman D (1991) *Practical Statistics for Medical Research*. T.J. Press (Padstow) Ltd.: Padstow, Cornwall

Bates TE, Williams SR, Gadian DG, Bell JD, Small RK and Iles RA (1989) $^{1}$H-NMR study of cerebral development in the rat. *NMR Biomed* **2:** 225–229

Bruhn H, Frahm J, Gyngell ML, Merboldt KD, Hänicke W, Sauter R and Hamburger C (1989) Non-invasive differentiation of tumours with use of localised H-1 MR spectroscopy *in vivo*. Initial experience in patients with cerebral tumours. *Radiology* **172:** 541–548

Burger PC, Grahmann FC, Bliestle A and Kleihues P (1987) Differentiation in the medulloblastoma – a histological and immunohistochemical study. *Acta Neuropathol* **73:** 115–123

Cerdan S, Parrilla R, Santoro J and Rico M (1985) $^{1}$H-NMR detection of cerebral myo-inositol. *FEBS Lett* **187:** 167–172

Cruz-Sanchez FF, Rossi ML, Hughes JT, Esiri MM and Coakham HB (1989) Medulloblastoma. *Acta Neuropathol* **79:** 205–210

Cudkowitz M and De La Monte SM (1989) Histogenesis and cell lineage analysis of the medulloblastomas. *J Neurol Sci* **94:** 221–229

Florian C-L, Preece NE, Bhakoo KK, Williams SR and Noble M (1995) Cell type-specific fingerprinting of meningioma and meningeal cells by $^{1}$H-NMR spectroscopy. *Cancer Res* **55:** 420–427

 © Cancer Research Campaign 1997

Florian C-L, Preece NE, Bhakoo KK, Williams SR and Noble M (1996) Characteristic metabolic profiles revealed by $^{1}$H nuclear magnetic resonance spectroscopy for three types of human brain and nervous system tumours. *NMR Biomed* **8**: 253–264

Frahm J, Michaelis T, Merboldt KD, Bruhn H, Gyngell ML and Hänicke W (1990) Improvements in localised $^{1}$H-NMR spectroscopy of human brain. Water suppression, short echo times and 1ml resolution. *J Magn Reson* **90**: 464–473

Friedman HS, Burger PC, Bigner SH, Trojanowski JQ, Wikstrand CJ, Halperin EC and Bigner DD (1985) Establishment and characterization of the human medulloblastoma cell line and transplantable xenograft D283 Med. *J Neuropathol Exp Neurol* **44**: 592–605

Fung KM, Chikaraishi DM, Suri C, Theuring F, Messing A and Albert DM (1994) Molecular phenotype of Simian-virus 40 large T-antigen-induced primitive neuroectodermal tumours in four different lines of transgenic mice. *Lab Invest* **70**: 114–124

Gill SS, Thomas DGT, Van Bruggen N, Gadian DG, Peden CJ, Bell JD, Cox IJ, Menon DK, Iles RA, Bryant DJ and Coutts GA (1990) Proton NMR spectroscopy of intracranial tumours: in vivo and in vitro studies. *J Comput Assist Tomogr* **14**: 497–504

Jacobsen PF, Jenkyn J and Papadimitriou JM (1985) Establishment of a human medulloblastoma cell line and its heterotransplantation into nude mice. *J Neuropathol Exp Neurol* **44**: 472–485

Kleihues P, Burger PC and Scheitauer BW (1993) The new WHO classification of brain tumours. *Brain Pathol* **3**: 255–268

Koller KJ, Zaczek R and Coyle JT (1984) *N*-acetyl-aspartyl-glutamate: regional levels in rat brain and the effects of brain lesions as determined by a new HPLC method. *J Neurochem* **43**: 1136–1142

Kotitschke K, Jung H, Nekolla S, Haase A, Bauer A and Bogdahn U (1994) High-resolution one-and two-dimensional 1H-MRS of human brain tumour and normal glial cells. *NMR Biomed* **7**: 111–120

Lindroth P and Mopper K (1979) High performance liquid chromatographic determination of subpicomole amounts of amino acids by precolumn fluorescence derivation with o-phthaldialdehyde. *Anal Chem* **51**: 1667–1674

Miyake M and Kakimoto Y (1981) Developmental changes of *N*-acetyl-L-aspartic acid *N*-acetyl-α-aspartylglutamic acid and β-citryl-L-glutamic acid in different brain regions and spinal cords of rat and guinea pig. *J Neurochem* **37**: 1064–1067

Pietsch T, Scharman T, Fonatsch C, Schmidt D, Ockler R, Freihoff D, Albrecht S, Zeltzer P and Riehm H (1994) Characterization of five new cell lines derived from human primitive neuroectodermal tumours of the central nervous system. *Cancer Res* **54**: 3278–3287

Preece NE, Baker D, Butter C, Gadian DG and Urenjak J (1993) Experimental allergic encephalomyelitis raises betaine levels in the spinal cord of strain 1 guinea pigs. *NMR Biomed* **6**: 194–200

Remy C, Arus C, Ziegler A, Sam Lal E, Moreno A, Le Fur Y and Decorps M (1994) In vivo, ex vivo, and in vitro one- and two-dimensional nuclear magnetic resonance spectroscopy of an intracerebral glioma in rat brain: assignment of resonances. *J Neurochem* **62**:166–179

Rorke LB, Gilles FH, Davis RL and Becker LE (1985) Revision of the WHO classification of brain tumours for childhood brain tumours. *Cancer* **56**: 1869–1886

Smith PK, Krohn RI, Hermanson GT, Mallia AK, Gartner FH, Provenzano MD, Fujimoto EK, Goeke NM, Olson BJ and Klenk DC (1985) Measurement of protein using bicinchoninic acid. *Anal Biochem* **150**: 76–85

Sze DY and Jardetzky O (1990) Determination of metabolite and nucleotide concentrations in proliferating lymphocytes by $^{1}$H-NMR of acid extracts. *Biochim Biophys Acta* **1054**: 181–197

Trojanowski JQ, Tohyama T and Lee VMY (1992) Medulloblastomas and related primitive neuroectodermal brain-tumours of childhood recapitulate molecular milestones in the maturation of neuroblasts. *Mol Chem Neuropathol* **17**: 121–135

Trojanowski JQ, Fung KM, Rorke LB, Tohyama T, Yachnis AT and Lee VMY (1994) In vivo and in vitro models of medulloblastomas and other primitive neuroectodermal brain tumours of the childhood. *Mol Chem Neuropathol* **21**: 219–239

Urenjak J, Williams SR, Gadian DG and Noble M (1992) Specific expression of *N*-acetylaspartate in neurons, oligodendrocyte-type-2 astrocytes and immature oligodendrocytes in vitro. *J Neurochem* **59**: 55–61

Urenjak J, Williams SR, Gadian DG and Noble M (1993) Proton nuclear magnetic resonance spectroscopy unambiguously identifies different neural cell types. *J Neurosci* **13**: 981–989

© Cancer Research Campaign 1997